# From Ad Hoc to Association to Academy: Developmental Milestones Over 48 Years for AGHE

**DOI:** 10.1093/geroni/igaa057.1809

**Published:** 2020-12-16

**Authors:** Lisa Hollis-Sawyer, Lydia Manning

## Abstract

The Academy for Gerontology in Higher Education began as the Ad Hoc Committee on the Development of Gerontology Resources in 1972. The next year the name Association for Gerontology in Higher Education (AGHE) was selected. In 1974 AGHE held a general meeting of its membership in conjunction with the annual meeting of the Gerontological Society of America in Portland Oregon. The first annual meeting was held in 1975 in Madison, Wisconsin. The relationship with GSA has evolved over the last four decades leading to the present Academy. The first presentation will focus on the issues of gerontology and higher education that emerged in the early years and continue to be a challenge today. The second presentation will look at the future of AGHE in its new relationship with GSA. The third presentation will focus on the Academy in the present discussing current challenges facing academic gerontology in today’s world including the development of AGHE’s basic-competency guidelines and the emergence of measuring student learning outcomes and their role in program review and evaluation. The fourth presentation will examine the evolving discussion of standards in evaluating gerontological and geriatric programming. Geriatric Education Interest Group Sponsored Symposium.

